# Community Care in Reach: Mobilizing Harm Reduction and Addiction Treatment Services for Vulnerable Populations

**DOI:** 10.3389/fpubh.2020.00501

**Published:** 2020-09-25

**Authors:** Craig Regis, Jessie M. Gaeta, Sarah Mackin, Travis P. Baggett, Joan Quinlan, Elsie M. Taveras

**Affiliations:** ^1^Kraft Center for Community Health, Community Health Improvement, Massachusetts General Hospital, Boston, MA, United States; ^2^Boston Health Care for the Homeless Program, Boston, MA, United States; ^3^AHOPE Boston Needle Exchange Program, Boston Public Health Commission, Boston, MA, United States; ^4^Division of General Internal Medicine, Massachusetts General Hospital, Boston, MA, United States; ^5^Division of General Academic Pediatrics, Mass General Hospital for Children, Boston, MA, United States

**Keywords:** mobile health, health access, substance use disorder, addiction, community health, harm reduction

## Abstract

Opioid overdoses killed 47,600 people in the United States in 2017. Despite increasing availability of office-based addiction treatment programs, the prevalence of opioid overdose is historically high and disproportionately affects vulnerable populations, including people experiencing homelessness. Despite availability of effective treatment, many at greatest risk of death from overdose experience myriad barriers to care. Launched in 2018, the Community Care in Reach mobile health initiative uses a data-driven approach to bring harm reduction and medication for opioid use disorder directly to those at highest risk of near-term death. Proof-of-concept results suggest that mobile addiction services may serve as a model for expanding access to addiction care for the most vulnerable.

## Introduction

In 2017, 47,600 people died from an opioid overdose in the United States—a 140% increase since 2000 (1, 2). In Massachusetts, nearly six lives are lost every day to an opioid-related overdose (3). While 60% of patients who receive medication for opioid use disorder achieve sustained remission, nearly 80% of people with opioid use disorder do not receive treatment (4, 5). Vulnerable populations, including people experiencing homelessness or who are vulnerably housed, are at particularly high risk for overdose death with rates 20 times higher than in the general population (3). Despite the existence of effective treatments and services, people with opioid use disorder (OUD) do not receive treatment due to barriers caused by stigma within healthcare, recovery, and correctional settings, complex co-occurring health and social conditions, and federal regulations limiting buprenorphine and methadone access. Additionally, few office-based addiction treatment programs offer a full range of harm reduction services that can help mitigate risks to the health and safety of people with OUD. Thus, the opioid overdose crisis is an epidemic of poor access to care.

To address this public health crisis, in January 2018 The Kraft Center for Community Health at Massachusetts General Hospital partnered with Boston Health Care for the Homeless Program (BHCHP) and the Boston Public Health Commission (BPHC) to launch an innovative mobile health initiative targeting opioid overdose “hotspots” in Greater Boston. The initiative, known as the Community Care in Reach program, aimed to increase access to harm reduction services, addiction treatment, and primary care by bringing on-demand services directly to marginalized individuals at highest risk of near-term death from a drug overdose. The program deploys caregivers to overdose “hotspots,” providing a non-traditional combination of clinical and harm reduction services (Table 1) (6).

**Table 1:** Addiction, primary care, and harm reduction services provided by the Community Care in Reach mobile addiction program.

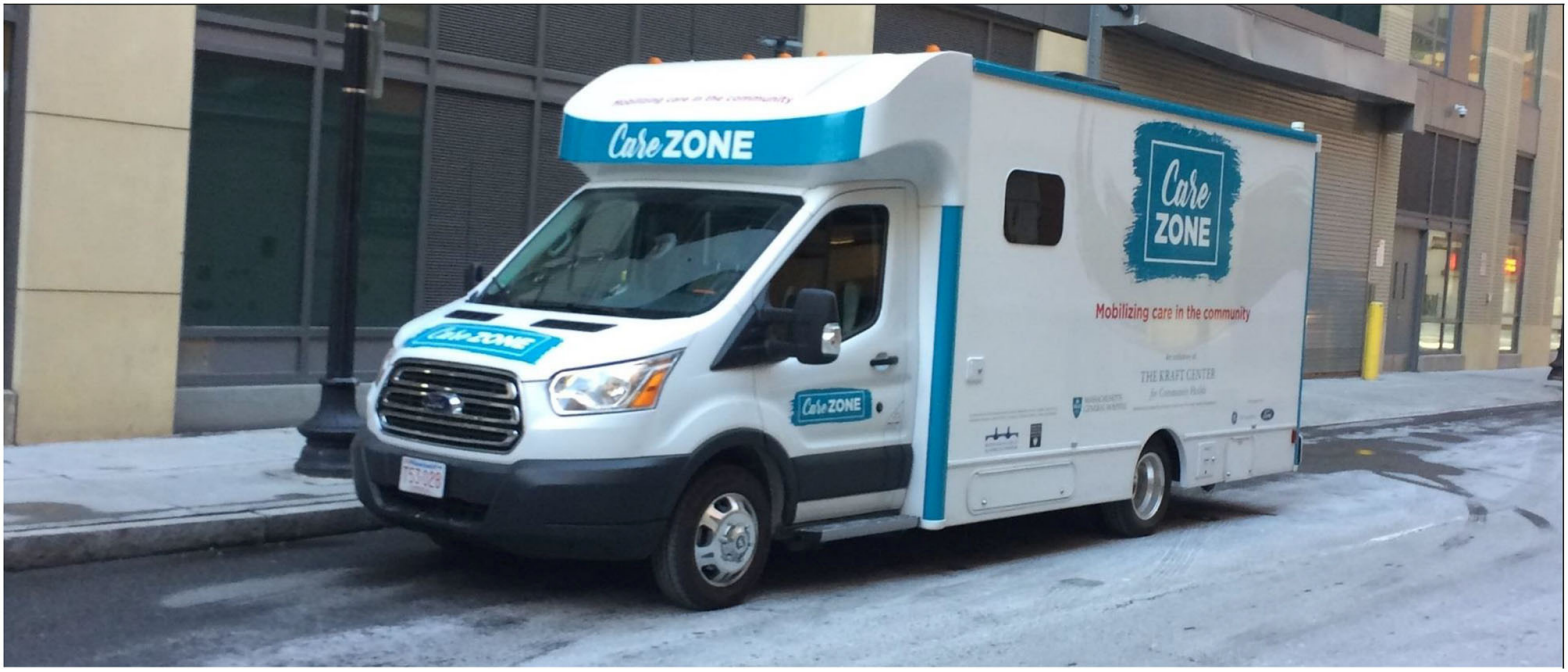
**PROGRAM SERVICES**
**Addiction care**
• Medication for Opioid Use Disorder (MOUD), including: ◦ ***Buprenorphine*** – prescribed on the van with an observed induction on the first day of treatment and ongoing monitoring for response and diversion via mucosal toxicology ◦ ***Naltrexone*** – oral or injection, prescribed and administered directly on the van ◦ ***Methadone*** – requires referral from the van to licensed outpatient methadone programs • Inpatient Detoxification – calls made from the van and transportation arranged • Pre- and post-exposure prophylaxis (PEP and PrEP) for HIV • Referrals for behavioral therapies
**Primary care**
• Screenings – for HIV/STI, Hepatitis C, tuberculosis, cancer • Vaccinations – influenza, hepatitis A & B, and any other vaccinations • Wound Care – management of soft tissue infections caused by injection drug use • Tele-behavioral health consults • Referrals for specialty care
**HARM REDUCTION SERVICES**
• Syringe exchange and collection • Naloxone distribution and training • Counseling on reducing risk when using drugs • Drug residue testing

## Context

Community Care in Reach was designed for people with OUD not currently engaged in care and at highest risk for near-term death from overdose. The overall purpose of the program is to increase access to OUD treatment and harm reduction services. The program also serves as a bridge to long-term, community-based care and recovery services.

Though the model may be iterated to meet the needs of various settings and environments, the program was developed for an urban setting: Boston, Massachusetts. The program launched in January 2018 and has continued through March 2020.

**Figure 1:**
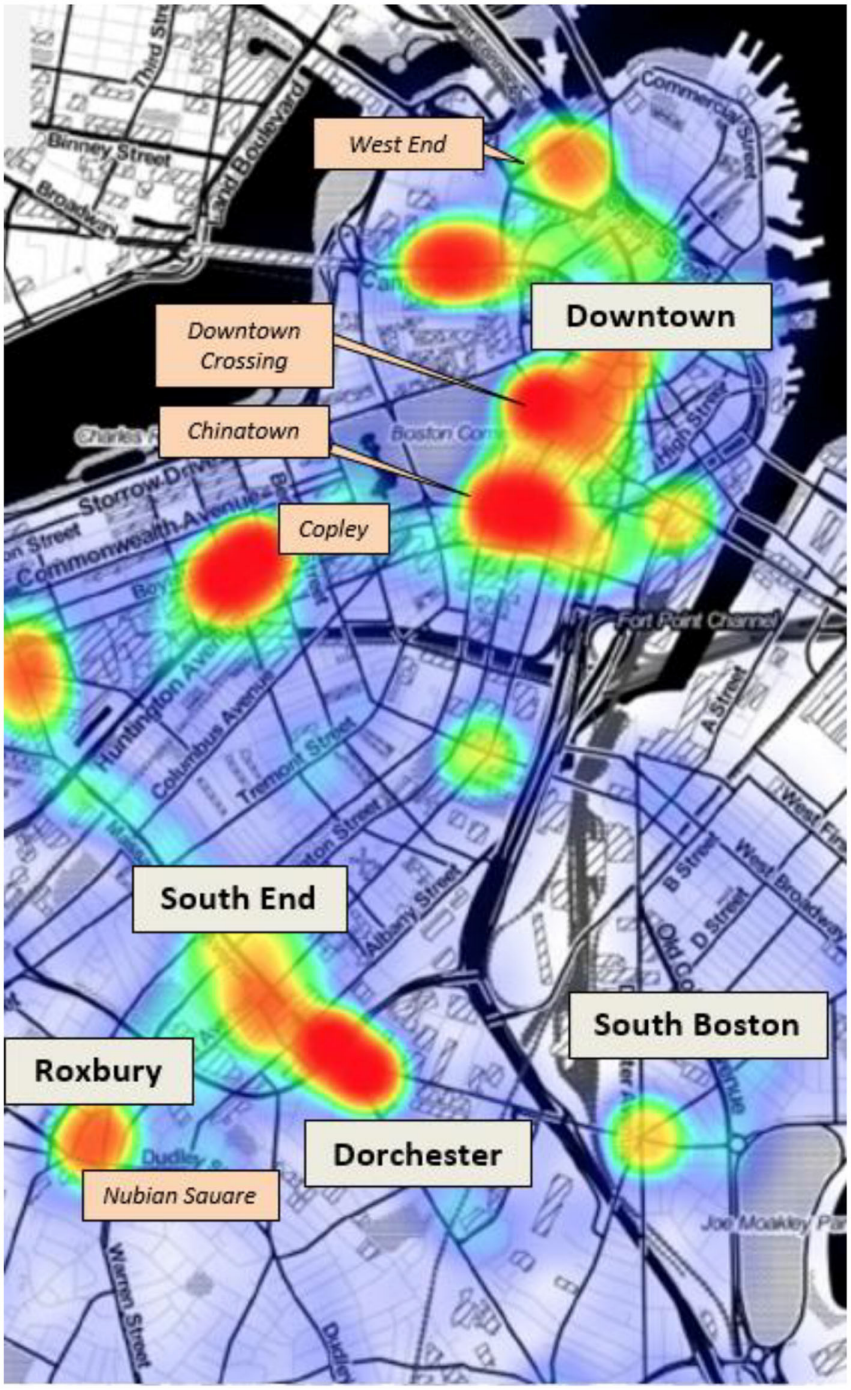
Narcotic-related incident Hotspots in Boston, 2016. Data from Boston Emergency Medical Services, Boston Public Health Commission.

In July 2017, and in collaboration with the Boston Public Health Commission and the Massachusetts Department of Public Health, we used narcotic-related incident data from BPHC to map overdoses across Boston (See map in Figure 1). Neighborhoods with consistently high rates of opioid overdose incidents with relatively few existing addiction services or street outreach work were prioritized as pilot sites.

We then conducted an extensive community engagement process to share the proposed model with stakeholders, solicit feedback, and coordinate proposed services while establishing channels of communication through which to share program updates and assess concerns during implementation. Engagement efforts began with neighborhood liaisons from Boston City Hall's Office of Neighborhood Services (ONS) and involved meetings with community health centers, neighborhood associations, law enforcement, business coalitions, homeless service organizations, and other local stakeholders. It was through discussions with ONS that the first two sites in the West End and Roxbury neighborhoods of Boston were selected based on high overdose rates indicated in narcotic-related data, relatively fewer existing street-level services addressing addiction, and local willingness to serve as a pilot site for a novel mobile addiction services program.

## Programmatic Elements

We commissioned the build of a 24′ mobile medical unit large enough for a private, fully-equipped exam room, yet small enough to easily navigate the streets of Boston with no commercial driver's license. The van is also equipped with a sink, a wheelchair lift, a small reception area, an electric-powered awning, a bike rack, and two refrigerators for vaccine and food storage. The vehicle runs on diesel fuel and a lithium battery powers clinical operations in the rear of the vehicle. The vehicle is plugged in at the conclusion of clinical operations to recharge the battery.

We launched a 10-month pilot in January 2018, deploying the van initially to two Boston neighborhoods and expanding to four by May 2018. The mobile health team that staffs each clinical session includes one buprenorphine-waivered clinician with expertise in addiction and caring for homeless populations and three harm reduction specialists from the City of Boston's syringe service program, working in pairs to conduct street outreach and offer harm reduction services and equipment to people either known to be living with OUD or exhibiting signs of addiction.

BPHC's Access Harm Reduction, Overdose Prevention and Education (AHOPE) program staff lead the harm reduction component of Community Care in Reach with the goal of providing non-judgmental support based on the needs of people actively using drugs by engaging with them directly on the street. AHOPE staff offer anonymous risk reduction options including supplies such as naloxone kits for overdose reversal, unused syringes, and personal hand-held biohazard containers for used syringes. Other harm reduction activities include naloxone training, disposal of used syringes, HIV/HCV testing, education around safer injection practices, and referrals to various substance use treatment facilities depending on the needs and preferences of each individual. Engagement with people who use drugs by AHOPE staff is conducted via street outreach, with Boston Health Care for the Homeless Program (BHCHP) health care providers often accompanying them (6).

BHCHP primary care physicians with training in addiction medicine lead the clinical care component of Community Care in Reach. Each physician has attained the federally-required certifications to prescribe buprenorphine for patients with OUD. The goal of the clinical component is to expand access to addiction treatment by providing ultra-low threshold services including medications for OUD (MOUD) with primary and preventive care tailored specifically to address the health needs of people actively using drugs. Most clinical encounters occur in the mobile medical unit which was custom-built for the program. The unit includes a single exam room and exam chair as well as a small reception and triage area separated by a pocket door. Other amenities include a sink, a medical refrigerator, and cabinets where medical supplies are stored (6).

Aggregate outreach data were collected and reported by the AHOPE staff weekly to the Boston Health Care for the Homeless Institute for Research, Quality & Policy in Homeless Health Care. BHCHP used a standardized template to chart clinical encounters their electronic health record (6). Full demographics of patients and services provided during the pilot period are shown in Table 2.

**Table 2:** Baseline characteristics and services provided to Community Care in Reach patients in the City of Boston, January 16, 2018–December 31, 2019.

Patient Demographics*	***N* = 119**	**Services provided**	
**Demographics**		**Outreach**	
Age, Years, Mean (SD)	38.4 (12.0)	Contacts	9,808
		Syringes distributed	96,601
Gender, *N* (%)		Naloxone kits distributed	2,956
Female	39 (32.8)		
Male	79 (66.4)	**Patients**	
Transgender	1 (0.8)	Clinical encounters	1336
Race/ethnicity, *N* (%)		Unique patient visits	328
Hispanic	12 (10.1)	Patients w/>2 visits	150
Non-Hispanic white	63 (52.9)		
Non-Hispanic black	11 (9.2)	**Medications prescribed**	
Other/unknown	33 (27.7)	Buprenorphine prescriptions	854
Housing status, *N* (%)		Buprenorphine patients	164
Street	81 (68.1)		
Shelter	12 (10.1)	Vaccines given**	
Doubled Up	10 (8.4)	Influenza	44
Housed	8 (6.7)	Pneumonia	54
Other	8 (6.7)	TdAP	23
		Hepatitis A	97
**Medical conditions**		Hepatitis B	88
Hepatitis C, *N* (%)	50 (42.0)	HPV	1
HIV, *N* (%)	7 (5.9)	Meningococcal	17
**Psychiatric conditions**		Tests performed**	
Depression, *N* (%)	22 (18.5)	HIV	68
Anxiety, *N* (%)	10 (8.4)	Hepatitis C	42
PTSD, *N* (%)	17 (14.3)		
Bipolar disorder, *N* (%)	12 (10.1)		
**Substance use disorders**			
Alcohol use disorder, *N* (%)	7 (5.9)		
Drug use disorders, *N* (%)			
Opioid	89 (74.8)		
Cocaine	17 (14.3)		
Marijuana	9 (7.6)		
Sedative/hypnotic	7 (5.9)		
Stimulant	6 (5.0)		

* *Data collected 1/6/2018-11/16/2018*.

** *These data are underestimates due to limitations related to extraction of these variables from the HER*.

In addition to scheduled weekly clinics, the model affords the program the flexibility to respond to emergent needs in other areas of the community. We continue to monitor overdose trends and population health data and can deploy the van to communities experiencing increased overdose rates or acute public health crises. In May 2018, Community Care in Reach led a rapid response to reported infectious disease exposures in the Fenway neighborhood of Boston, offering HIV testing, prophylaxis, and vaccines, along with clinical care for OUD and harm reduction services. The response resulted in an added clinical site where the program continues to provide weekly support to a network of individuals otherwise disconnected from health care.

As of December 31, 2019, the Community Care in Reach team made 9,098 contacts with people with OUD, distributing a total of 96,601 syringes and 2,956 naloxone kits. Clinicians had 1,336 clinical encounters with 328 unique patients and provided 854 buprenorphine prescriptions to 164 unique patients. Clinicians also provided 68 HIV tests and 42 hepatitis C tests and administered 324 vaccinations for influenza, pneumonia, TdAP, hepatitis A, hepatitis B, HPV, and meningitis.

## Evaluation

We used quantitative and qualitative methods to evaluate the pilot program. All evaluation procedures were approved by the Partners Health Care Human Research Committee. For quantitative analyses, we extracted coded clinical and harm reduction encounter data from the electronic health records and compiled the data for analysis. We also conducted a concurrent qualitative study to elicit perceptions from selected patients of the services they received through Community Care in Reach and to provide additional context for the findings of the quantitative component. We generated a list of potential candidates for interviews across sites based on factors such as frequency of van visits, types of van visits, anticipated availability, and anticipated willingness to participate. All potential subjects (those pre-identified and those not) had the clinician's approval for recruitment purposes. Clinicians also asked these patients if it is okay for someone to speak with them about a research study they may be eligible for. We only approached patients with permission from both the clinician and patient, approaching them after their Community Care in Reach visit so as not to interfere with the triage and intake process before the visit. The clinician alerted study staff to those who may be in distress and thus inappropriate to take part in the study. After describing the study and obtaining and documenting verbal consent from eligible patients, the interviewer proceeded to find a secure, private, safe space near the van to conduct the interview. From September through December 2018, we conducted semi-structured interviews with seven patients across all program service sites. All participants ranged in age from 26 to 61 and had accessed program services at some capacity, including five who were receiving addiction treatment through a Community Care in Reach medical provider (6).

Quantitative analyses demonstrated the existing demand for mobile addiction services, with the team logging over 3,800 contacts with people who use drugs and 308 clinical encounters in the first 10 months. Trends also showed a ramping up of services over time as the program became more established and built trust in the community. Results also demonstrate that buprenorphine treatment can succeed in a mobile setting, as 47 unique patients received prescriptions and all 28 undergoing follow-up toxicology screenings yielded one or more buprenorphine-positive result and 19 yielded at least 1 opioid-negative results. Records also indicate the program served as an important entry point for engaging new patients with over half (55%) being new to BHCHP services.

Qualitative interviews demonstrated high acceptability of the model by people who often experience stigma in traditional healthcare settings. Participants reported the value in compassionate care that is both predictable and flexible. Representative domains and participant quotes are provided in Table 3. Participants confirmed a high demand for medical services paired with proactive street outreach. Convenience was identified as a major benefit of the model. Interviews also pointed to successful engagement methods, highlighting relationship building over time and describing staff as “compassionate” and “helpful.” Proposed improvements included expanding hours, locations, and outreach strategies to engage with a larger patient population, yet another indication of the high demand and acceptability for the program.

**Table 3:** Representative quotes of community care in reach patients in the City of Boston, January 16, 2018–December 31, 2019^a^.

**Qualitative interview themes**	**Representative patient quotations**
Patient experiences with substance use	“Yeah, I've had a lot of people overdose… In the last three years, my best friend … passed away from an overdose, and then when my brother overdosed with me there …, that made me see it differently. And experiencing an overdose myself when a loved one had to be there for me, and realizing their perspective it's a lot more serious than you think it is, it's scary. It's very scary.” – Community Care in Reach patient
Safe and legal buprenorphine prescriptions	“To get suboxone. It's safer, legal. Health-wise, I feel so much better. I feel more secure, I feel more stable, getting back on a regimen, luckily given by a doctor, better than buying them on the street and maybe not taking them properly. Safety wise it's so much better.” – Community Care in Reach patient
Compassionate, non-judgmental medical care	“I would describe them as pleasant, helpful, and compassionate… It's a good thing, it shows people do care, when you are hopeless and down and out and you feel like no one cares and you can come here, and you realize people do care.” – Community Care in Reach patient
Accessible, reliable, and convenient medical care	“That it wasn't like… that it didn't have specific timed appointments, just go and show up. The people that work on the van were all cool.” – Community Care in Reach patient “Yeah, I can walk here, and they show up when they say they are going to be here. I like the crew; the crew is good. The van is proper, its clean. But its more economical, more convenient for me, because I'm having trouble with T-passes and trying to get to any other doctor, or proper facility is challenging. Money-wise isn't is great right now so it helps out, it's very convenient.” – Community Care in Reach patient
Negative experiences with traditional medical care	“They have always been very negative about what my problems are or about what my issues are. They just don't let me, they just want to do what they think is what I need. And I have problems that I want to talk about and they just don't want to listen. And these people on the van are willing to listen and give me the time get help.” – Community Care in Reach patient
Relationship building is key	“No. It was good that they walked around and let everyone know they were here and what their abilities were, you know, like what their reason was for being out here.” – Community Care in Reach patient
Suggested improvements	“I know if you were closer to up here [Massachusetts Avenue and Melnea Cass Boulevard intersection] you would get more people. If you were to get the van right outside the Engagement Center you would have more people than you would know what do to with.” – Community Care in Reach patient “Well maybe they should put out some flyers. Because I heard it word of mouth. I don't see anything around Dudley station saying well we have a van…” – Community Care in Reach patient

a *Data originally presented in (6)*.

Our evaluation efforts focused on assessing reach and accessibility of Community Care in Reach, though ongoing analyses will provide larger context for program impact and effectiveness. Ongoing evaluation efforts include assessing trends in overdose rates in services areas as reported by Boston EMS, a prospective cohort study assessing changes in health care utilization in program patients vs. a comparable control group, and a cost effectiveness analysis to identify potential costs averted through reduced emergency department visits and inpatient intakes for complications from drug injury.

## Discussion

Launched in 2018, the intended goal of the Community Care in Reach program was to provide targeted addiction treatment, harm reduction services, and primary care directly to individuals at highest risk for near-term death from drug overdose. The program combines two components not typically offered in the same setting: clinical care and harm reduction services, which are both provided via a mobile clinical van and street outreach (6). Early results indicate success in expanding access to health care for people with OUD and high levels of demand for and acceptability of the mobile medical model. Currently, the model requires outside public or private funding to sustain services, as many of the addiction services beside the encounters with a clinician are not billable, including harm reduction services, supplies, and activities. Ongoing evaluation will determine to what extent the program results in decreased economic burden on the healthcare system by reducing hospital stays or emergency department visits, potentially making it a cost-effective investment for state and federal funders. The program has been recognized as a best practice in Massachusetts where the State's Harm Reduction Commission highlighted early successes in its March 2019 report (7)^1^. MDPH is also expanding mobile addiction programming statewide by awarding four agencies, including Community Care in Reach partner BHCHP, 5-year grants to implement mobile health initiatives to replicate the success of Community Care in Reach (8)^2^.

Though syringe service programs and medications for opioid use disorder are infrequently co-located, Community Care in Reach provides a novel integration of these addiction services in a mobile setting. This patient-centered solution flips the concept of “the doctor will see you now,” and employs a data-driven approach to bring proven interventions directly to populations at highest risk of near-term death who are currently not accessing healthcare in traditional settings due to numerous barriers. A mobile model also affords care teams the flexibility to shift resources when data indicate emerging needs or trends.

Community Care in Reach's early successes indicate an existing demand for mobile addiction services and establish a blueprint for successful buprenorphine treatment in a non-traditional setting. Findings demonstrate high demand and acceptability among the target population following a ramp-up period to boost engagement and reach capacity. Future considerations include integrating HIV and hepatitis C treatment, and ongoing evaluation will further analyze the larger health and economic implications of this innovative, highly adaptable model.

## Conceptual Constraints

All addiction services provided by the program are well-established, evidence-based interventions, though stigma may fuel community resistance to addiction services. Early and ongoing community engagement is critical to retaining support from local stakeholders. Because Community Care in Reach targets high risk populations with many barriers to care, loss to follow-up is a major challenge. Outreach workers employ multiple strategies, including outreach via phone and word of mouth to reconnect with patients who have fallen out of care. An ongoing challenge of the program has been bridging patients to office-based addiction treatment programs where they do not have existing relationships. We continue to collaborate with local healthcare facilities to establish more seamless referrals for patients who indicate a desire for long term treatment services.

## Conclusion

Curbing the rates of overdose death will require innovative approaches beyond what is delivered in traditional models of healthcare delivery. Too many individuals never make it through the doors of our brick-and-mortar institutions to access the addiction care and services they need.

Community Care in Reach mobilizes effective, evidence-based services to bring care directly to people in greatest need. Early results indicate this is a highly acceptable model among the target population and can serve as an entry point into care for those who are not currently engaged. The model effectively flips the concept of “the doctor will see you now” on its head and demonstrates the potential impact of meeting people where they are, both figuratively and literally.

## Data Availability Statement

The raw data supporting the conclusions of this article will be made available by the authors, without undue reservation.

## Ethics Statement

The studies involving human participants were reviewed and approved by Partners Health Care Human Research Committee. The ethics committee waived the requirement of written informed consent for participation.

## Author Contributions

ET, JQ, JG, and SM conceived of the program. ET oversaw implementation of the program and oversaw editing of the manuscript. JG and SM led program operations and contributed to analysis. TB led the analysis. CR led the writing. All authors contributed to the article and approved the submitted version.

## Conflict of Interest

TB receives royalties from UpToDate for authorship of a topic review on homeless health care. The remaining authors declare that the research was conducted in the absence of any commercial or financial relationships that could be construed as a potential conflict of interest.

## Footnotes

^1^https://www.mass.gov/doc/harm-reduction-commission-report-3-1-2019/download

^2^https://www.mass.gov/news/dph-announces-14-million-annually-for-new-mobile-addiction-services
